# The Effect of a Multivitamin and Mineral Supplement on Immune Function in Healthy Older Adults: A Double-Blind, Randomized, Controlled Trial

**DOI:** 10.3390/nu12082447

**Published:** 2020-08-14

**Authors:** Mary L. Fantacone, Malcolm B. Lowry, Sandra L. Uesugi, Alexander J. Michels, Jaewoo Choi, Scott W. Leonard, Sean K. Gombart, Jeffrey S. Gombart, Gerd Bobe, Adrian F. Gombart

**Affiliations:** 1 Linus Pauling Institute, Department of Biochemistry & Biophysics, Oregon State University, Corvallis, OR 97331, USA; m.l.fantacone@gmail.com; 2 Linus Pauling Institute, Department of Microbiology, Oregon State University, Corvallis, OR 97331, USA; malcolm.lowry@oregonstate.edu; 3 Linus Pauling Institute, Oregon State University, Corvallis, OR 97331, USA; sandra.uesugi@oregonstate.edu (S.L.U.); alexander.michels@oregonstate.edu (A.J.M.); jaewoo.choi@oregonstate.edu (J.C.); scott.leonard@oregonstate.edu (S.W.L.); skgombart@gmail.com (S.K.G.); jsgombart@gmail.com (J.S.G.); 4 Linus Pauling Institute, Department of Animal & Rangeland Sciences, Oregon State University, Corvallis, OR 97331, USA; gerd.bobe@oregonstate.edu

**Keywords:** vitamin C, zinc, vitamin D, multivitamin, placebo, randomized clinical trial, immune, illness

## Abstract

Older adults are at increased risk for vitamin and mineral deficiencies that contribute to age-related immune system decline. Several lines of evidence suggest that taking a multi-vitamin and mineral supplement (MVM) could improve immune function in individuals 55 and older. To test this hypothesis, we provided healthy older adults with either an MVM supplement formulated to improve immune function (Redoxon^®^ VI, Singapore) or an identical, inactive placebo control to take daily for 12 weeks. Prior to and after treatment, we measured (1) their blood mineral and vitamin status (i.e., vitamin C, zinc and vitamin D); (2) immune function (i.e., whole blood bacterial killing activity, neutrophil phagocytic activity, and reactive oxygen species production); (3) immune status (salivary IgA and plasma cytokine/chemokine levels); and (4) self-reported health status. MVM supplementation improved vitamin C and zinc status in blood and self-reported health-status without altering measures of immune function or status or vitamin D levels, suggesting that healthy older adults may benefit from MVM supplementation. Further development of functional assays and larger study populations should improve detection of specific changes in immune function after supplementation in healthy older adults. Clinical Trials Registration: ClinicalTrials.gov #NCT02876315.

## 1. Introduction

Worldwide vitamin deficiencies play a primary etiological role in global disease burden [1]. About 35% of older adults in the United States, Canada and Europe are deficient in one or more micronutrients [2,3]. This is especially the case for vitamin C [4,5], zinc [6], and vitamin D [7,8,9]. These deficiencies may contribute to age-related decline of the immune system [10,11], which is most often characterized by increased levels of inflammation, reduced innate immune function and reduced T-cell function [12,13].

Although many nutrients play vital roles in the immune system, numerous studies highlight the importance of vitamin C, vitamin D, and zinc [14,15]. Vitamin C is important for neutrophil phagocytosis, motility, reactive oxygen species (ROS) generation, antimicrobial activity and monocyte locomotion [16,17,18,19,20,21]. Zinc sufficiency alters numbers and function of neutrophils, monocytes, natural killer T cells and B cells [22,23] and is an important mineral for neutrophil and monocyte chemotaxis [24]. Vitamin D is critical for both innate and adaptive immune function and it is required for induction of the cathelicidin antimicrobial peptide gene in activated monocytes/macrophages [25,26,27].

Inadequate vitamin C intake is associated with an increased risk of pneumonia and severe respiratory infection [21,28]. Regular supplementation with vitamin C, when taken prior to onset, reduces the risk of contracting the common cold [29]. Low intake of zinc, prevalent among older adults, correlates with impaired immune function [30,31]. Although only some randomized controlled trials have found that zinc supplementation reduces the number of infections in older adults [32], zinc lozenges significantly lowered the mean duration of cold symptoms [33,34,35,36,37]. Low serum 25-hydroxyvitamin D [25(OH) vitamin D] is linked to a higher risk of acute respiratory tract infections [38]. Overall, meta-analysis of randomized controlled trials indicate daily supplementation with vitamin D may reduce the risk of upper respiratory tract infections [38]. Supplementation studies with combinations of these micronutrients have also suggested efficacy in immune function [39,40].

Since multiple nutrients support immune function, older adults may benefit from multivitamin and mineral (MVM) supplements [41]. Generally regarded as safe and readily available over-the-counter, dietary supplements have been used with few significant side effects in clinical studies [14]. Although conflicting and contradictory studies exist, there is evidence suggesting that dietary supplementation with a combination of immunity-related micronutrients supports immune function and reduces risk or severity of infection [14]. Indeed, targeted supplementation with these vitamins and minerals may provide additional protection at doses higher than the U.S. recommended dietary allowance (RDA) [15]. 

Thus, we hypothesized daily MVM supplements containing immune essential micronutrients including vitamin C, vitamin D, and zinc could improve immune cell function in an older population. To test this hypothesis, we conducted a double blind randomized placebo-controlled trial on healthy adults 55–75 years old with Redoxon^®^ Vita Immune (VI) MVM supplements. To assess immune function, we evaluated the changes to whole blood bacterial killing activity against *Staphylococcus aureus*, phagocytic activity, production of reactive oxygen species (ROS) and other immune parameters. We expected that the combination of vitamins and minerals found in Redoxon^®^ VI would increase the bactericidal activity in blood from supplemented individuals as compared to the placebo.

## 2. Materials and Methods

### 2.1. Study Design

This was a single-center, two-armed, parallel, randomized, double-blinded study designed to determine the impact of daily oral intake of an MVM, Redoxon^®^ VI (Table 1), on immune function in healthy, older adults after 12 weeks of supplementation. This trial was registered on August 23, 2016 prior to inclusion of the first participant (ClinicalTrials.gov # NCT02876315). The full protocol is available upon request. All subjects gave their informed consent before they participated in the study, which was conducted at the Linus Pauling Institute at Oregon State University (OSU) according to the principles of the Declaration of Helsinki and approved by the Institutional Review Board at OSU (Study #7531, 08/04/2016).

The primary study outcome was change in bacterial killing by whole blood between baseline and 3 months of supplementation. Secondary outcomes were changes in neutrophil phagocytic activity and ROS production and inflammatory cytokine levels between baseline and 3 months of supplementation. Additional endpoints were self-reported health status, and changes in blood mineral and vitamin status (i.e., zinc, vitamin C and vitamin D) and immune status (salivary IgA) between baseline and 3 months of supplementation.

### 2.2. Study Participants

Eligible men and women between 55 and 75 years old were recruited from the OSU campus, the surrounding community and the LIFE Registry at the Center for Healthy Aging Research at OSU. After an initial telephone screen, eligible participants were invited to schedule an appointment at the Clinical Research Center at the Linus Pauling Institute. At the screening visit, the study nurse reviewed the purpose of the study, study activities, and the consent form. Upon receiving written informed consent, the study nurse collected health history, height, weight, heart rate, blood pressure, and a blood spot sample for vitamin D measurement. Only healthy individuals with serum 25-(OH) vitamin D levels ≥25 nmol/L (10 ng/mL) were invited to participate in the study.

For inclusion in the trial, individuals agreed to limit intake of salmon, shellfish, herring, sardines, beef, poultry dark meat, lamb, and liver to one 4-ounce serving per week and to limit intake of citrus fruits and fruit juices to two servings per day for 3 weeks prior to and throughout the study. They also agreed to stop taking multivitamins, supplements containing zinc, vitamins C and D, and food/beverage products supplemented with zinc and vitamins C and D for 3 weeks prior to and throughout the study.

Individuals excluded from participation were those that had smoked tobacco, e-cigarettes or any other substance 3 months prior to or during the study; had a surgical procedure 2 months prior to or planned during the study; had gastrointestinal surgery or procedures; consume more than two alcoholic drinks per day; had cardiovascular, kidney or liver disease; gastrointestinal, endocrinological, or immunological disorders; organ or tissue transplant; inflammatory skin conditions; were diagnosed with autoimmune disease or HIV positive status; a history of cancer less than 5 years prior; Stage II hypertension (either systolic blood pressure >159 mm Hg or diastolic blood pressure >99 mm Hg); BMI < 18.5 or >29.9. Other exclusion criteria: individuals with hypervitaminosis A, D, or hypercalcemia; a reported dietary intake of zinc >15 mg/day; participation in another dietary or clinical study within 2 months prior to this study; recent history of UV therapy or tanning; reported allergies (medications, foods, or seasonal) or allergic asthma. Individuals currently taking or using any medications that may alter immune response, possibly interact with vitamin D, alter absorption of vitamin D [24,25], or are not recommended to be taken simultaneously with an MVM were also excluded.

### 2.3. Method of Assigning Participants to Intervention Groups

Upon approval for entry into the study, the study statistician assigned participants to one of two intervention groups using a randomized block design. Participants were blocked by sex (male, female) and age groups (55–61, 62–68, 69–75) into six homogeneous groups. Within their block, participants were assigned to one of two intervention groups (Table 2) using a random number generator in Microsoft Excel. The study statistician instructed the study nurse to provide appropriate film-coated tablets to participants. The participants and study staff were not aware of the identity of treatment assignments during the duration of the study. The code linking participants with intervention was lifted after endpoint data for all participants were collected and analyzed. The total study duration was approximately 12 months (9 December 2016–28 November 2017), as enrollment of participants was staggered as opposed to simultaneous.

### 2.4. Intervention and Sample Collection

The study timeline was as follows: Dietary restrictions began 3 weeks before intervention. At time 0, participants returned to the study site for baseline measurements: 51 mL blood and 1 mL saliva samples were collected by the study nurse. Following specimen collection, participants received their first 6-weeks supply of supplements/placebo and a Supplement and Illness Diary. In this diary, subjects were instructed to record their daily supplement intake and a brief survey adapted from the Wisconsin Upper Respiratory Symptom Survey-21 [42] to note any illness symptoms, including a severity rating of illness. 

Participants returned to the study site on week 6. Remaining supplements and their completed diary were collected. The study nurse inquired about adverse events and collected a 10 mL blood sample. Subjects received their second 6-week supply of supplements and another diary. This process was repeated on week 12, except that a 51 mL blood sample and a 1 mL saliva sample were obtained and participation ended.

### 2.5. Whole Blood Killing Assay

The whole blood killing assay was modified from a previously described protocol [43]. Briefly, *Staphylococcus aureus* (Rosenbach strain) bacteria were pelleted, washed twice, and were diluted to 1.0 × 10^6^ CFU/mL in PBS (without Ca^2+^ and Mg^2+^), and 20 µL was immediately mixed with 180 µL of freshly drawn peripheral whole blood in a sterile heparinized 2 mL round-bottom micro centrifuge tube. Samples were mixed at 37 °C on a rotary shaker and at 0, 120 and 240 min post-infection, and lysed in sterile deionized water (1800 µL) by pipetting and vortexing. Then 100 µL of lysate from each time point as well as several 10-fold serial dilutions were spread onto 10 cm diameter bacterial growth plates. Following an overnight incubation at 37 °C, plates were photographed and colony-forming units (CFUs) enumerated.

### 2.6. Phagocytic Activity, Total ROS Generation, Cytokine and Salivary IgA Measurements

Phagocytic activity was measured by quantifying the uptake of pHrodoTM Red *Escherichia coli* (LifeTechnologies, Carlsbad, CA, USA) at times 0, 15, 30, and 45 min by fluorescence-activated cell sorting (FACS). Only those bacteria phagocytosed by neutrophils would fluoresce as the dye signal increases in intensity as pH decreases in the phagosome. Total ROS production was measured by FACS using dihydrorhodamine (eBioscience, San Diego, CA, USA) after stimulation with phorbol-12-myristate-13-acetate (PMA).

Cytokine levels were measured using a Luminex 200 and the Inflammation 20-Plex Human ProcartaPlex^TM^ Panel as instructed by the manufacturer (Life Technologies). An ELISA was used as instructed by the manufacturer (Abcam, Cambrige, MA, USA) to determine salivary IgA (sIgA) levels.

### 2.7. Vitamin C, D and Zinc Assessment

Vitamin C levels were measured in the Linus Pauling Institute’s Analytical Services Core Laboratory by HPLC coupled to electrochemical detection as described previously [44]. Serum samples were sent to ARUP Laboratories (Salt Lake City, UT, USA) for measurement of zinc levels. Spot blood and serum samples were sent to ZRT Labs (Beaverton, OR, USA) for measurement of 25(OH) vitamin D levels.

### 2.8. Statistical Analysis

The study was designed to show a statistically significant (*p* ≤ 0.025) 40% increase in whole blood bacterial killing activity, neutrophil phagocytic activity, and ROS production, respectively, with 80% power. The power calculation was based on a coefficient of variation of 40% and 21 healthy, senior adults per treatment group.

Statistical analyses were performed using SAS version 9.4 software (SAS Institute, Cary, NC, USA). The primary statistical endpoints were changes in bacterial killing by whole blood and neutrophil function (phagocytosis and superoxide production) between baseline and 12 weeks of supplementation. Since we had repeated measures within participants, we used a repeated measures in time design in PROC MIXED for blood variables and measures of immune function. Fixed effects in the model were treatment (placebo, Redoxon^®^ VI), weeks (0, 12), and their interaction as well as the blocking variables age, group and gender. The repeated measures within participants were modeled with a compound symmetry variance-covariance matrix. To adjust the degrees of freedom for repeated measures, the Kenward–Rogers approximation for degrees of freedom was used. The primary contrasts were treatment by week interaction and the comparisons between weeks 12 and 0 within placebo participants (n = 21) and within Redoxon^®^ VI participants (n = 21). For data without baseline values and for comparison of baseline values, PROC GLM for quantitative data and PROC GLIMMIX with a negative binomial distribution link for the categorical illness data was used. Fisher’s exact test for binary data such as gender or percentage ill was used. To evaluate seasonal effects on vitamin level and reported illness days and severity, we tested for effect modification by season of participation and did not detect statistically significant effect modification (results not shown). All tests were two-sided. Significance of group differences was determined to be at *p* ≤ 0.05. We did not adjust for multiple comparisons.

## 3. Results

### 3.1. Study Participants

**Figure 1:**
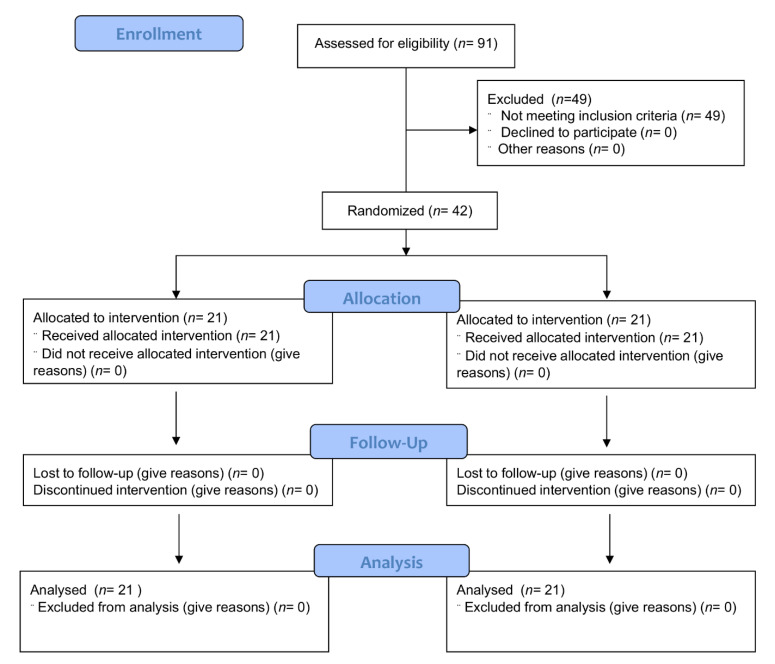
CONSORT flow diagram of recruitment, allocation, follow-up and analysis.

We screened 91 individuals through phone interviews. Of these, serum 25(OH) vitamin D levels were measured in 43 participants. One individual was excluded for overt vitamin D deficiency (level <10 ng/mL); the remaining 42 individuals (94% identified as Caucasian, 2% American Indian/Alaskan Native, 2% Asian and 2% more than one race) were randomized to the two study arms (21 per arm, Figure 1). The baseline characteristics of the participants are described in Table 3. 

Overall, the groups were well matched. A wide range of plasma vitamin C levels measured in the participants was noted, suggesting a wide range of normal vitamin C intake from diet. Although all participants had serum zinc levels in the normal range, two individuals had serum zinc levels ≥ 100 µg/dL suggesting high dietary intake of zinc.

### 3.2. Compliance

All participants completed the study. Based on records of supplement intake, reported compliance was high with 30/42 at 100%. The compliance of the remaining 12 participants ranged from 84–99%. 

### 3.3. Response to Supplementation

**Figure 2:**
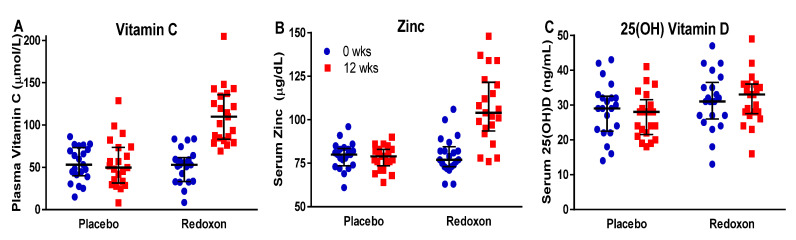
The effect of MVM supplementation on circulating levels of vitamin C, zinc and vitamin D in placebo and Redoxon^®^ VI arm participants at baseline and 12 weeks post-supplementation. Compared with placebo, Redoxon^®^ VI increased (**A**) plasma vitamin C (*p* < 0.0001), (**B**) serum zinc (*p* < 0.0001), and (**C**) did not alter serum 25(OH) vitamin D concentrations (*p* = 0.15). The graph shows individual participant values in blue at baseline and red at 12 weeks; black horizontal lines show the median and interquartile range.

To determine if MVM supplementation increased levels of immune-modulating micronutrients in the blood of participants, we measured plasma vitamin C, serum 25(OH) vitamin D and serum zinc before and after supplementation (Table 4). The results show that after 12 weeks of intervention, plasma vitamin C (Figure 2A) increased 126% in the Redoxon^®^ VI arm (change from baseline +59.7 ± 6.4 µmol/L, *p* < 0.0001), but remained unchanged in the placebo arm (change from baseline +0.5 ± 6.4 µmol/L; *p* = 0.94). Similarly, serum zinc (Figure 2B) increased 43% in the Redoxon^®^ VI arm (change from baseline +27.2 ± 3.7 µg/dL; *p* < 0.0001) but remained unchanged in the placebo arm (change from baseline −1.2 ± 3.7 µg/dL; *p* = 0.75; Figure 2B). For 25(OH) vitamin D (Figure 2C), no significant changes were observed in the Redoxon^®^ VI arm (change from baseline +0.6 ± 1.1 ng/mL; *p* = 0.61) and the placebo arm (change from baseline −1.8 ± 1.1 ng/mL; *p* = 0.12).

Zinc, vitamin C, and 25(OH) vitamin D levels in blood were positively correlated with each other at week 12 (zinc with vitamin C: r = +0.73, *p* < 0.001; zinc with vitamin D: r = +0.47, *p* = 0.002; vitamin C with vitamin D: r = +0.36, *p* = 0.02). In summary, the MVM supplement improved zinc and vitamin C status but did not significantly affect vitamin D status. 

### 3.4. Whole Blood Killing of S. aureus

**Figure 3:**
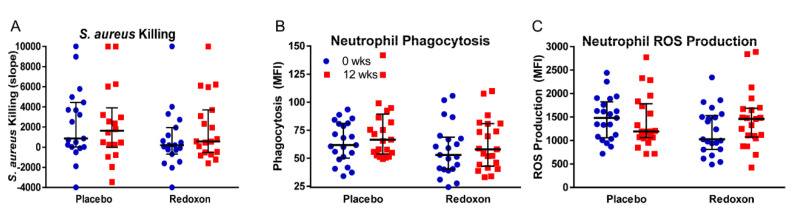
The effect of MVM supplementation on immune function in placebo and Redoxon^®^ VI arm participants at baseline and 12 weeks post-supplementation. Placebo and Redoxon^®^ VI participants did not differ in (**A**) whole blood *S. aureus* killing (*p* = 0.29), (**B**) neutrophil phagocytosis (*p* = 0.53) or (**C**) neutrophil ROS production (*p* = 0.13). The graph shows individual participant values in blue at baseline and red at 12 weeks; black horizontal lines show the median and interquartile range.

As a primary functional outcome of immune function, we measured the potential of whole blood to kill introduced amounts of *S. aureus*. No significant treatment differences (*p* = 0.29) were detected between baseline and after 12 weeks of supplementation within placebo (change from baseline: −296 ± 897, *p* = 0.74) and MVM arms (change from baseline: +1057 ± 897 ∆MFI, *p* = 0.25) (Figure 3A, Table 4). Whole blood killing showed a modest inverse relationship with plasma vitamin C levels (r = −0.37; *p* = 0.02), but no association with serum levels of zinc (r = −0.12; *p* = 0.48) or vitamin D (r = −0.17; *p* = 0.31). Among indicators of immune function (neutrophil phagocytosis, neutrophil superoxide production, and whole blood killing), there were no significant correlations between the observed data from baseline to 12 weeks, suggesting they were measuring different aspects of neutrophil activity. The strongest correlation was between phagocytosis and whole blood killing: r = +0.28, *p* = 0.099.

### 3.5. Neutrophil Phagocytosis

To determine if MVM supplementation improved immune function by improved clearance of foreign particles, we measured neutrophil phagocytosis using commercially available fluorescence-tagged, heat-killed *E. coli* as described above. No significant treatment differences (*p* = 0.53) were detected between baseline and after 12 weeks of supplementation within placebo (change from baseline: +9.6 ± 5.0 MFI, *p* = 0.06) and MVM arms (change from baseline: +5.2 ± 4.9 MFI, *p* = 0.30) (Figure 3B, Table 4). Since data collection occurred over the course of a 45 min exposure, we looked at the individual time points for the experiments. Increases from baseline (0 min time point) were observed at the 15 min time point, which were significant in both treatment arms (placebo: +6.1 ± 3.0 MFI; *p* = 0.047; Redoxon: +7.8 ± 3.0 MFI; *p* = 0.01) (data not shown). At week 12, for both arms of the study, we found a modest inverse relation between phagocytosis and plasma levels of vitamin C (r = −0.31; *p* = 0.0499), but no association with serum levels of zinc (r = −0.08; *p* = 0.63) or vitamin D (r = +0.02; *p* = 0.91).

### 3.6. Neutrophil Superoxide Production

To determine if MVM supplementation improved immune function by improved respiratory burst, we also measured neutrophil oxidant production using dihydrorhodamine as described above as a proxy for superoxide production. Measurement of ROS production serves as a potential indicator of neutrophil microbicidal activity [45]. We maximally stimulated the superoxide-generating capacity of the whole blood cells with PMA. No significant treatment differences (*p* = 0.13) were detected between baseline and after 12 weeks of supplementation within placebo (change from baseline: −81 ± 151 MFI, *p* = 0.59) and MVM arms (change from baseline: +253 ± 151 MFI, *p* = 0.10) (Figure 3C, Table 4). Neutrophil oxidant production showed a modest inverse relationship with serum vitamin D (r = −0.32; *p* = 0.04) but no association with serum zinc (r = +0.01; *p* = 0.96) or plasma vitamin C (r = −0.01; *p* = 0.97). 

### 3.7. Salivary IgA and Serum Inflammatory Cytokine Levels

Changes in salivary IgA levels or serum levels of cytokines and chemokines can indicate immune and inflammation status, and may serve as an indicator of a change in nutrient availability. Salivary IgA concentrations decreased in Redoxon^®^ VI arm participants from baseline to week 12 (from 1.95 ± 0.26 to 1.34 ± 0.26 ng/mL, *p* = 0.02; Table 5), whereas no changes were observed in the placebo group (from 1.44 ± 0.23 to 1.50 ± 0.23 ng/mL, *p* = 0.80). Although this change resulted in significant treatment differences (*p* = 0.047), it is worth noting that salivary IgA levels were equal in both groups after 12 weeks. We did not observe significant changes in serum levels of cytokines and chemokines when comparing the baseline versus 12 weeks for the placebo or MVM arms (Table 5).

### 3.8. Reported Illness

To monitor adverse events and potential health impacts of MVM supplementation, we instructed the participants to record any illness symptoms and rate severity of illness as described above. In general, we found that MVM supplementation decreased self-reported disease severity and duration, but not incidence (Table 6). More specifically, placebo and Redoxon^®^ VI arms did not differ in the percentage of participants that reported illness; however, MVM supplementation decreased the number of days ill by nearly 70% from 6.43 ± 1.71 in the placebo arm to 2.29 ± 0.77 in the Redoxon^®^ VI arm (*p* = 0.02).

Participants in the Redoxon^®^ VI arm usually experienced on most days ill only very mild symptoms (62%), whereas placebo arm participants experienced mild to severe disease symptoms on most days ill (60%), with 10 of 13 reporting three or more symptoms. This observed increase in days ill and severity is reflected in a statistically-significant four-fold increase in “days ill x severity” in the placebo arm (*p* = 0.008).

A modest, inverse correlation of days ill x severity was observed with serum levels of zinc, but not plasma levels of vitamin C or serum levels of vitamin D at week 12 (days ill x severity with zinc: r = −0.40; *p* = 0.01; vitamin C: r = −0.25; *p* = 0.12; vitamin D: r = −0.21; *p* = 0.18). Days ill x severity was not correlated with measures of immune function (days ill x severity with neutrophil phagocytosis: r = −0.11; *p* = 0.50; neutrophil superoxide production: r = +0.12; *p* = 0.47; whole blood killing: r = +0.11; *p* = 0.52). The participants did not report any adverse events from either the placebo or MVM.

## 4. Discussion

Since studies on both single-nutrient and some MVM supplements indicate efficacy in treating and reducing the risk of infections [14], there is good rationale to test the effect these supplements have on specific aspects of the immune system. There is also a clear rationale to test the effect of these supplements in older adults, who are not only at risk for decreased immune function, including a loss of neutrophil function [46], but multiple vitamin and mineral shortfalls in the diet [41]. Although neutrophil function did not appear to change in the study, we present data showing an MVM supplement that is high in several redox-active and immunomodulatory micronutrients, and increases blood levels of both vitamin C and zinc in study participants. In turn, these changes in nutrient levels were associated with a significant decline in reported illness in the MVM treatment group.

Neutrophils from older adults show a decreased ability to kill *S. aureus* [46], suggesting a therapeutic window for select micronutrients; therefore, we focused on neutrophil function as a primary outcome in this study. Prior studies with zinc and vitamin C would suggest that demonstrable change in neutrophil function is possible with supplementation. Neutrophils from animals deficient in vitamin C display attenuated motility, phagocytosis, oxidative burst, and microbial killing compared to control animals or animals supplemented with vitamin C to reverse dysfunction [47,48,49]. Kiwifruit (about 260 mg/day of vitamin C) increased chemotaxis and oxidative burst in neutrophils isolated from the blood as compared to baseline in 14 volunteers [50]. Chelation of free zinc (Zn^2+^) in vitro also impairs neutrophil function including chemotaxis, phagocytosis and oxidative burst [51]. In vivo, decreased zinc levels impair natural killer cell activity, phagocytosis by macrophages and neutrophils and oxidative burst activity [52]. In patients with inflammatory rheumatic diseases, a 60-day treatment with 45 mg/day zinc improved neutrophil phagocytic activity [53].

By contrast, the effects of micronutrients on neutrophil function are not always consistent. A systematic review identified 16 RCTs that assessed the effect of vitamin C supplementation on neutrophil function as primary or secondary outcomes only showed a positive effect in 44% of the studies [54]. In healthy individuals, consumption of vitamin C at 375 mg/day for 10 weeks did not affect either phagocytosis or oxidative burst and the same was observed in children consuming up to 100 mg/day over eight weeks [55,56]. Furthermore, consumption of gold kiwifruit (about 360 mg/day vitamin C) by older adults did not affect neutrophil phagocytosis [57].

In the current study, individuals in the MVM arm consumed 1 g/day vitamin C and 10 mg/day zinc for 12 weeks. We observed significant increases in the blood levels of both micronutrients in the MVM arm and not the placebo arm, but we did not observe statistically significant changes in phagocytosis or oxidative burst in whole blood cells. We also did not observe statistically significant changes in killing of *S. aureus* by whole blood. Therefore, our findings did not support our hypothesis that an MVM can improve neutrophil functional outcomes.

There may be several reasons for the apparent failure in an MVM supplement to affect neutrophil activity. First, although we asked our participant population to refrain from high zinc and vitamin C intake, their baseline blood measures show varied amounts of these two micronutrients. No participant suffered from inadequate or deficient zinc levels at baseline, while two participants had zinc levels that were above 100 µg/dL. At least half of the participants had plasma vitamin C values that were considered near optimal (>50 µM). Therefore, it is possible that the MVM supplement had a limited effect in this apparently well-nourished population. In future studies, we should incorporate prescreening to enroll participants with low vitamin C and zinc status [54]. Secondly, the assays we used to assess neutrophil activity may need further optimization using recently described improvements [58,59]. Detecting ROS production using dihydrorhodamine is subject to artifactual amplification of fluorescent intensity via a redox-cyclin mechanism and it is not specific for superoxide detection, as a variety of reactive oxygen and nitrogen species can produce fluorescence after reacting with the molecule [60]. Thirdly, it is possible that neutrophil activity is a less than ideal indicator of nutrient status, and that other immune markers might be more sensitive to changes in diet or supplement intake [61]. Lastly, the study size was small. With more participants, we may have reached significance for some of the functional assays.

We did observe a statistically significant decrease in severity and length of reported illnesses in treatment versus placebo. In prior studies with patients that were suffering from the common cold, improvements with a short-term treatment combining zinc with high-dose vitamin C were noted [40]. Authors of both randomized controlled trials and systematic reviews of these trials concluded that zinc could reduce cold duration [33,34,35,36,37]. In this study, we treated participants for 3 months and monitored self-reported illness during this period. We observed significant increases in both zinc and vitamin C in the MVM arm and it was associated with a significant decrease in duration and severity of illness (about 3-fold for duration and about 3–6 fold for severity) compared with the placebo arm. Although this was not one of our primary outcomes, we note it as an intriguing observation that is indicative of clinical relevance. However, because our functional assays for neutrophil function did not reach statistical significance, we cannot conclude that the difference involves modulation of the immune system. The results from this study will help design future long-term studies. Selection of different or additional immune biomarkers to assess immune modulation and increasing participant numbers may improve our ability to detect functional changes in immune function due to nutritional supplementation [61].

We observed a modest inverse association of vitamin C with phagocytosis and whole blood killing in both arms, suggesting that with higher levels of vitamin C there was a decrease in phagocytosis and whole blood killing. We also observed a similar modest inverse association for vitamin D and ROS production. While this is counterintuitive, it is possible that the increased days of illness and severity observed in the placebo arm participants confounded the functional tests. On the other hand, increased zinc levels were modestly associated with a decrease in days ill x severity. 

We observed a statistically significant decrease in sIgA levels in the MVM arm from baseline levels that were higher at week 0 than in the placebo arm. The final mean levels for the MVM arm were similar to the baseline and 12 weeks levels in the placebo arm. With decreased sIgA levels, we might expect an increase in illness/infection, but we postulate that the decrease was not biologically significant.

## 5. Conclusions

MVM supplementation for 12 weeks significantly increased zinc and vitamin C levels in the study participants. We did not observe statistically significant changes in immune outcomes, but we observed a statistically significant decrease in reported length and severity of illnesses in the MVM arm versus the placebo arm. Despite the technical limitations of functional assays, our findings support further research to test our hypothesis that MVM supplementation can improve immune outcomes in older adults. The findings from this study are sufficient to inform our design for future studies on supplements and immune function.

## Figures and Tables

**Table 1:** Composition of Redoxon^®^ VI for Two Film Coated Tablets.

Active Ingredients	Units	Amount	RDA	UL
Vitamins				
Vitamin A	µg	700	700	3000
Vitamin D	IU	400	600	4000
Vitamin E	mg	45	15	1000
Vitamin B6	mg	6.6	1.3	100
Folate	μg	400	400	1000
Vitamin B12	μg	9.6	2.4	-
Vitamin C	mg	1000	75	2000
Trace Elements				
Iron	mg	5	18	45
Copper	mg	0.9	0.9	10
Zinc	mg	10	8	40
Selenium	µg	110	55	400
Other Ingredients				
Microcrystalline cellulose, magnesium stearate, hydroxylpropylmethylcellulose, hydroxypropylcellulose hypromellose, titanium dioxide, microcrystalline cellulose, iron oxide yellow, sodium croscarmellose, and talc.				

Abbreviations: RDA (Recommended Dietary Allowance); UL (Tolerable Upper Intake Level).

**Table 2:** Intervention groups.

Arms	Assigned Interventions
Redoxon^®^ VI Comparison is made within participants prior to and after treatment and to placebo arm; *n* = 21	Supplement: two Redoxon^®^ VI tablets, 1X/day, oral; 12 weeks
Placebo Comparison is made within participants prior to and after placebo treatment and to the Redoxon^®^ VI supplementation arm; *n* = 21	Supplement: two tablets of inert materials, 1X/day, oral; 12 weeks

**Table 3:** Baseline characteristics for participants (mean ± STD).

Characteristics	Placebo (*n* = 21)	MVM (*n* = 21)	*P*-Value
Age (years)	63 ± 5	64 ± 5	0.78
Gender (self-identified)			1
Female	16	15	
Male	5	6	
BMI (kg/m^2^)	26 ± 3	25 ± 3	0.28
Systolic blood pressure (mmHg)	129 ± 13	122 ± 10	0.07
Diastolic blood pressure (mmHg)	81 ± 7	77 ± 6	0.08
Heart Rate (bpm)	66 ± 8	65 ± 8	0.66
Plasma Vitamin C (µmol/L)	54 ± 20	52 ± 20	0.68
Serum 25(OH) vitamin D (ng/mL)	29 ± 8	31 ± 8	0.89
Serum Zinc (µg/dL)	79 ± 8	80 ± 11	0.34
Whole Blood *S. aureus* Killing (slope)	+1671 ± 3856	+298 ± 3129	0.25
Neutrophil Phagocytosis (MFI)	64 ± 18	58 ± 23	0.33
Neutrophil ROS Production (MFI)	1471 ± 460	1201 ± 495	0.07

Abbreviations: BMI (body mass index); MVM (multi-vitamin and mineral, Redoxon^®^ VI); STD (standard deviation).

**Table 4:** Micronutrient levels and immune function measures in placebo and Redoxon^®^ VI participants at baseline and 12 weeks post-supplementation.

	Placebo		Redoxon^®^ VI			*P*-Differences		
	Wk 0	Wk 12	Wk 0	Wk 12	SEM	PL	RDX	Treat
Micronutrients:								
Vitamin C	51.5	51.9	48.9	108.6	6.2	0.94	<0.01	<0.01
Zinc	78.9	77.7	79.1	106.4	2.9	0.75	<0.01	<0.01
25(OH) Vit D	27	25.2	29.9	30.5	1.8	0.12	0.61	0.15
Immune Function:								
Whole Blood								
Killing	1588	1292	234	1292	877	0.74	0.25	0.29
Neutrophils:								
Phagocytosis	64.3	73.9	57.4	62.5	5.3	0.06	0.3	0.53
ROS Prod.	1546	1465	1250	1503	118	0.59	0.1	0.13

Plasma vitamin C levels are in µmol/L; serum zinc levels are in µg/dL; serum 25(OH)vitamin D levels are in ng/mL; whole blood *S. aureus* killing activity are expressed as a slope; and neutrophil phagocytosis and ROS production levels are in mean fluorescent intensity (MFI). The averages are least-squares means. The standard error of the mean (SEM) indicated is the largest of the four SEM. P-differences are the contrasts between week (wk) 12 and 0 in the placebo arm (PL) and in the Redoxon^®^ VI arm (RDX) and their interaction (Treat).

**Table 5:** Salivary IgA and cytokine/chemokine serum levels in placebo and Redoxon^®^ VI participants at baseline and 12 weeks post-supplementation.

Cytokine/Chemokine	Placebo		Redoxon^®^ VI			*P*-Differences		
	Wk 0	Wk 12	Wk 0	Wk 12	SEM	Plac	Red	Treat
Saliva:								
IgA	1.44	1.5	1.95	1.34	0.26	0.8	0.02	0.047
Serum:								
ICAM 1	26	29.2	39.3	39.3	19.6	0.21	0.99	0.39
IL 1A	2.68	2.69	3.33	1.87	0.83	0.99	0.07	0.17
IL 12p70	66.8	65.4	32.9	32	30.6	0.72	0.83	0.93

All measures are in pg/mL except for salivary IgA, ICAM 1, E-Sel, and P-Sel, which are in ng/mL. The averages are least-squares means. The standard error of the mean (SEM) indicated is the largest of the four SEM. P-differences are the contrasts between week (wk) 12 and 0 in the placebo arm (Plac) and in the Redoxon arm (Red) and their interaction (Treat).

**Table 6:** The effect of MVM supplementation on self-reported illness in placebo and Redoxon^®^ VI arm participants throughout the 12-week tablet consumption period.

	Placebo		Redoxon^®^ VI		
	Mean	SEM	Mean	SEM	P-Diff
Ill (% of participants)	65%	44%	48%	11%	0.35
Days ill	6.43	1.71	2.29	0.77	0.02
Days Level 1 severity	2.55	0.76	1.43	0.56	0.24
Days Level 2 severity	1.80	0.59	0.52	0.24	0.03
Days Level 3 severity	1.80	0.82	0.24	0.19	0.04
Days Level 4 severity	0.60	0.34	0.10	0.10	0.12
Days ill x Severity	13.95	4.63	3.57	1.25	0.008
Number of Symptoms	2.55	0.61	1.14	0.38	0.06

Level of severity: 1 = very mild; 2 = mild; 3 = moderate; 4 = severe.
